# Interruption of breast cancer care and importance of inter‐hospital cooperation during the COVID‐19 pandemic: A case report of advanced breast cancer in Fukushima, Japan

**DOI:** 10.1002/ccr3.6151

**Published:** 2022-08-03

**Authors:** Akihiko Ozaki, Yudai Kaneda, Yuki Senoo, Masahiro Wada, Tomohiro Kurokawa, Toyoaki Sawano, Masaharu Tsubokura, Tetsuya Tanimoto, Yoshiaki Kanemoto, Tomozo Ejiri, Hiroaki Shimmura, Norio Kanzaki

**Affiliations:** ^1^ Department of Breast and Thyroid Surgery Jyoban Hospital of Tokiwa Foundation Iwaki Japan; ^2^ Department of Gastrointestinal Tract Surgery Fukushima Medical University Fukushima Japan; ^3^ School of Medicine Hokkaido University Sapporo Japan; ^4^ Department of Breast Surgery Sano Kosei General Hospital Sano Japan; ^5^ Department of Surgery Jyoban Hospital of Tokiwa Foundation Iwaki Japan; ^6^ Department of Radiation Health Management Fukushima Medical University School of Medicine Fukushima Japan; ^7^ Department of Internal Medicine Jyoban Hospital of Tokiwa Foundation Iwaki Japan; ^8^ Department of Urology Jyoban Hospital of Tokiwa Foundation Iwaki Japan

**Keywords:** accessibility of health services, breast neoplasms, consultation, COVID‐19, referral, telemedicine

## Abstract

We experienced the case of a patient with advanced breast cancer who failed to receive comprehensive care despite regular video conferencing with her physician during the COVID‐19 pandemic, resulting in delayed detection of liver metastasis. Inter‐hospital collaboration is required to provide uninterrupted cancer care to those disproportionately affected by crises.

## Abstract 2

Few case‐based analyses have been conducted regarding the breast cancer patients who are disproportionately affected by the COVID‐19 pandemic. This case shows that patients with metastatic disease traveling from remote areas to see their physicians may need specific support so that they can receive uninterrupted care during the pandemic.

## 1 INTRODUCTION

Accurate and timely follow‐up is essential for breast cancer patients throughout their course of treatment. However, the novel coronavirus disease 2019 (COVID‐19) pandemic has readily hampered the continuation of breast cancer care in many parts of the world and Japan is no exception. In Japan, one of the first cases of COVID‐19 was detected in March 2020 in Tokyo, and from April to May 2020, the Japanese government declared a state of emergency in Tokyo and some other prefectures as the daily number of COVID‐19 cases increased exponentially. Consequently, the majority of the hospitals restricted out‐patient visits as an infection control measure against COVID‐19.^1^ In addition, some patients refrained from visiting hospitals to avoid contracting the virus.^2^ Indeed, COVID‐19 pandemic has been noted as a cause for interruption of such as radiation therapy during treatment for breast cancer.^3^ However, there is not enough information regarding breast cancer patients who are disproportionately affected by the COVID‐19 pandemic in terms of continuation of care.

Herein, we present the case of a patient with advanced breast cancer who resided in a remote area and could not undergo in‐person follow‐up. Despite routine remote video consultations, she did not receive comprehensive breast cancer care during the COVID‐19 pandemic, which resulted in delayed detection of liver metastasis.

## 2 CASE PRESENTATION

In January 2016, a 38‐year‐old premenopausal woman was diagnosed with right‐sided breast cancer at a medical institution in her hometown, Iwaki City, Fukushima prefecture, Japan. As she preferred receiving care in an urban setting, she was referred to a university hospital in Tokyo, Japan, which is approximately 200 km away from Iwaki City in February 2016. After extensive examinations, she was diagnosed with hormone receptor‐positive (estrogen and progesterone receptor‐positive [both 3b]), human epidermal growth factor receptor 2‐negative (2+ and dual‐color in situ hybridization equivocal) clinical T2N1M1 Stage IV breast cancer with asymptomatic multiple metastatic bone disease (right and left ilium, thoracic vertebras 4 and 7, and lumber vertebra 4) and symptomatic metastatic disease at a sternum.

In March 2016, endocrine treatment with tamoxifen, goserelin, and denosumab was initiated; however, it was switched to exemestane, goserelin, and denosumab in June 2017, after the computed tomography (CT) and breast ultrasonography showed an enlargement of the breast tumor and deterioration of bone metastases. Because local control could not be achieved using endocrine therapy alone, mastectomy and axillary dissection were performed in June 2019. The cancer subtype determined after the pathological analysis of surgical specimen was the same as that determined from the core needle biopsy performed for the initial diagnosis (estrogen and progesterone receptor‐positive [3b and 3a], human epidermal growth factor receptor 2‐negative [1+], Ki67 12%), and the same medical regimen was continued thereafter. In April 2020, she underwent a CT scan, and the results revealed no signs of recurrence. Her last in‐person visit to the university hospital was at the end of May 2020, when she received long‐acting goserelin (effective for 3 months). Even though the Japanese government lifted the state of emergency by the end of May 2020, she refrained from visiting the university hospital in Tokyo from Fukushima due to the persistent epidemic of COVID‐19 in Japan. Thereafter, her physician provided remote video consultations, namely in July and November 2020. Exemestane was prescribed virtually, and it was confirmed that her menses had not resumed. At this stage, her physician considered that a change in the prescribed regimen was not required because of the lack of evidence indicating disease progression and the difficulty in performing extensive follow‐up which is mandatory after starting a new treatment. She and her physician had been looking for a medical institution near her home where she could undergo follow‐up imaging regularly. However, they had been unable to find a suitable hospital for 6 months.

In November 2020, the patient was referred to our hospital. She had mild symptoms with an Eastern Cooperative Oncology Group Performance Status score of 1 just before visiting our hospital. Cancer antigen 15–3 and carcinoembryonic antigen levels were elevated to 87.2 U/ml and 7.6 ng/ml, respectively, and subsequent positron emission tomography in January 2021 revealed multiple liver metastases that were not detected in the examination by her previous doctor 9 months ago. Even though cyclin‐dependent kinase 4/6 inhibitors were available in Japan at the time, instead of these agents, chemotherapy with paclitaxel, bevacizumab, and denosumab was initiated in January 2021, because we assumed that her disease condition had been rapidly worsening. Follow‐up imaging performed in October 2021 revealed that liver metastases had shrunk in size.

## 3 DISCUSSION

This case highlights the factors due to which the COVID‐19 pandemic may impact the breast cancer patients disproportionately. These include residing in a remote area (or traveling a long distance to their physicians) and the presence of progressive disease. While similar situations may be taking place all around the world more than 2 years after the COVID‐19 pandemic was declared, it is important for each medical institution to predetermine what to do in the event of a pandemic for each patient.

In general, postoperative surveillance for early‐stage breast cancer patients includes annual mammography and other adjunctive investigations, such as ultrasonography and CT.^4^ On the contrary, for metastatic breast cancer patients or those experiencing disease recurrence, investigation of serum tumor marker levels and other imaging examinations performed every few months are recommended, depending on the patients' conditions.^5^ Regular follow‐up was particularly difficult for our patient because of the long‐distance travel required for each follow‐up visit and the fear of potentially contracting and spreading the infection. Indeed, traveling across prefectures was discouraged during the COVID‐19 pandemic in Japan.

In our case, considering the patient's condition, her primary physician understood the importance of performing a CT scan immediately, and the patient also made an effort to find a nearby hospital. However, it eventually took 7 months until she could visit our hospital, probably due to an increased burden on medical institutions brought on by the pandemic and their subsequent reluctance to accept new patients.

While the collaboration between medical institutions is well established, both in medical communities and society at large, its importance has been reiterated during the COVID‐19 pandemic. It has become increasingly difficult to find a hospital to refer patients to now that many hospitals are overburdened due to the pandemic and restricting the acceptance of new patients as a countermeasure. If our patient or her primary physician could have found a medical institution earlier, metastasis might have been detected in time. Particularly, given that a difficultly of long travel during the pandemic, it is important for urban medical institutions to prepare in advance how to deal with cases where it becomes difficult to properly treat patients from remote areas in their areas, such as by finding appropriate collaborators. While the information is lacking in other type of cancers, it cannot be deniable that similar situations may be occurring in their malignancies.

Nonetheless, we should take into account the situation surrounding Iwaki City, where this patient resides. There has long been a shortage of doctors in Iwaki, especially since the Great East Japan Earthquake in 2011, and the number of doctors has been decreasing significantly, by 18.1% from 2018 to 2020. As a result, another problem is that there are few doctors or hospitals in the area specializing in treating breast cancer patients. In this respect, if patients would reside in areas with a larger number of doctors, the patients may have continuously received a care after an interruption of care in institutions in urban areas.

This case also demonstrates the limitations of remote video consultations for cancer during the COVID‐19 pandemic. In fact, there has been a rapid increase in remote video consultations for patients with various diseases, including cancer.^6^ Similar to our case, some hospitals have started to practice telemedicine and providing drug prescriptions virtually to patients who cannot visit hospitals for various reasons during the COVID‐19, which was not widely practiced in Japan prior to the pandemic.^7^ However, without finding a nearby collaborating hospital, where the necessary examinations can be performed and treatments can be received when required, telemedicine should not be thoughtlessly implemented as a major strategy for provision of care, especially for patients with metastatic cancer or progressive disease.^8^

Considering that the COVID‐19 pandemic is occurring as successive waves in Japan, it is necessary to not only rely on the introduction of novel telemedicine technology but also to reform and adjust the traditional referral system for medical institutions, especially between urban and rural areas, so that it is suitable for the post‐pandemic era. Telemedicine can be a useful option; however, patients who have been receiving treatment at hospitals in urban areas should be able to continue their treatment in rural areas even when it becomes difficult for them to visit hospitals in urban areas.^9^

Therefore, it is important for medical institutions to take this opportunity to recognize the advantages and disadvantages of telemedicine. In general, remote video consultation is an effective method for patients with skin diseases that can be detected visually, or for follow‐up of patients with stable chronic conditions, such as hypertension.^10^ However, in cases like ours, the indication of telemedicine for patients requiring specialized diagnostic imaging on a regular basis is currently limited, and it is important to understand its limitations before selecting suitable patients. Further, telemedicine cannot be implemented reliably due to poor Internet connections or other problems and may not be used in low‐ and middle‐income countries as smoothly as in high‐income countries. In this sense, it is important to summarize the issues associated with telemedicine which have been clarified during the COVID‐19 pandemic in establishing a system that can address such issues as soon as possible.

## 4 CONCLUSIONS

In conclusion, we reported the case of a patient with advanced breast cancer who failed to receive comprehensive care despite regular video conferencing with her physician during the COVID‐19 pandemic, resulting in delayed detection of liver metastasis. Therefore, inter‐hospital collaboration is essential to provide accurate, timely, and uninterrupted follow‐up, which is essential for breast cancer treatment, to patients who are disproportionately affected by the crisis, specifically for recurrent or advanced cancer cases, and such collaboration is critical for the safe and efficient delivery of telemedicine to cancer patients during the COVID‐19 pandemic.

## AUTHOR CONTRIBUTIONS

Ozaki A, Kaneda Y, and Senoo Y drafted the manuscript. All authors made critical revisions of the manuscript and approved the final version of the manuscript.

## CONFLICT OF INTEREST

Ozaki A receives personal fees from MNES Inc, outside the submitted work. Tetsuya Tanimoto receives personal fees from MNES Inc, and Bionics co., ltd. outside the submitted work.

## CONSENT

A written consent has been obtained before submission and be made available to the publisher if requested.

## Data Availability

The data analyzed during the current study are available from the corresponding author on reasonable request.

## References

[1] Syed A , Kumari G , Kapoor A , et al. Impact of COVID‐19 on breast cancer management: a radiological prespective from a tertiary centre. Eur J Breast Health. 2021;17(2):180‐187. 33870119 10.4274/ejbh.galenos.2021.6379 PMC8025722

[2] Ozaki A , Sawano T , Saito H , Tanimoto T , Tsubokura M . Will initial consultation patterns among undiagnosed cancer patients be the same after this COVID‐19 pandemic? Experiences from the 2011 triple disaster in Fukushima, Japan. J Glob Health. 2020;10(2):020343. 10.7189/jogh.10.020343 PMC7648884 33214884

[3] Lee S , Heo J . COVID‐19 pandemic: a new cause of unplanned interruption of radiotherapy in breast cancer patients. Med Oncol. 2021;39(1):5. 34739633 10.1007/s12032-021-01604-9 PMC8569493

[4] Khatcheressian JL , Wolff AC , Smith TJ , et al. American Society of Clinical Oncology 2006 update of the breast cancer follow‐up and management guidelines in the adjuvant setting. J Clin Oncol. 2006;24(31):5091‐5097. 17033037 10.1200/JCO.2006.08.8575

[5] Stuart K , Brennan M , French J , Houssami N , Boyages J . Life after breast cancer. Aust Fam Physician. 2006;35(4):219‐224. 16642238

[6] Ozaki A , Tachibana K , Ohtake T . Challenges and future directions in breast cancer care in Fukushima prefecture in Japan: correspondence to “a survey on the current status of clinical resources for diagnosis and treatment of breast cancer in rural hospitals of the Tohoku region in Japan”. Breast Cancer. 2021;28(5):1163‐1164. 33880708 10.1007/s12282-021-01254-9 PMC8057288

[7] Bokolo AJ . Use of telemedicine and virtual care for remote treatment in response to COVID‐19 pandemic. J Med Syst. 2020;44(7):132. 32542571 10.1007/s10916-020-01596-5 PMC7294764

[8] Fouquet SD , Miranda AT . Asking the right questions‐human factors considerations for telemedicine design. Curr Allergy Asthma Rep. 2020;20(11):66. 32862299 10.1007/s11882-020-00965-x PMC7456356

[9] Chen Z , Sun S , Zhao W , et al. The impact of the declaration of the state of emergency on the spread of COVID‐19: a modeling analysis. Comput Math Methods Med. 2021;2021:8873059. 34426747 10.1155/2021/8873059 PMC8379385

[10] Ekeland AG , Bowes A , Flottorp S . Effectiveness of telemedicine: a systematic review of reviews. Int J Med Inform. 2010;79(11):736‐771. 20884286 10.1016/j.ijmedinf.2010.08.006

